# Long-term outcomes of omentum-preserving versus resecting gastrectomy for locally advanced gastric cancer with propensity score analysis

**DOI:** 10.1038/s41598-020-73367-8

**Published:** 2020-10-01

**Authors:** Yusuke Sakimura, Noriyuki Inaki, Toshikatsu Tsuji, Shinichi Kadoya, Hiroyuki Bando

**Affiliations:** 1 Department of Gastroenterological Surgery, Ishikawa Prefectural Central Hospital, Kanazawa, Ishikawa 920-8530 Japan; 2 Department of Surgery, Juntendo University Urayasu Hospital, Chiba, 279-0021 Japan

**Keywords:** Gastrointestinal diseases, Surgical oncology, Gastric cancer

## Abstract

Omentectomy is conducted for advanced gastric cancer (AGC) patients as radical surgery without an adequate discussion of the effect. This study was conducted to reveal the impact of omentum-preserving gastrectomy on postoperative outcomes. AGC patients with cT3 and 4 disease who underwent total or distal gastrectomy with R0 resection were identified retrospectively. They were divided into the omentum-preserved group (OPG) and the omentum-resected group (ORG) and matched with propensity score matching with multiple imputation for missing values. Three-year overall survival (OS) and 3-year relapse-free survival (RFS) were compared, and the first recurrence site and complications were analysed. The numbers of eligible patients were 94 in the OPG and 144 in the ORG, and after matching, the number was 73 in each group. No significant difference was found in the 3-year OS rate (OPG: 78.9 vs. ORG: 78.9, P = 0.54) or the 3-year RFS rate (OPG: 77.8 vs. ORG: 68.2, P = 0.24). The proportions of peritoneal carcinomatosis and peritoneal dissemination as the first recurrence site and the rate and severity of complications were similar in the two groups. Omentectomy is not required for radical gastrectomy for AGC.

## Introduction

Gastric cancer is the fifth most common cancer worldwide, with an estimated 952,000 new cases (7% of total cancer incidence) and 723,000 deaths (9% of total cancer mortality) in 2012^1^. Surgical resection of the primary tumour can lead to radical treatment; however, discussions about less invasive procedures and functional preservation for advanced gastric cancer (AGC) surgery have arisen recently^2–4^.

Omentectomy for gastric cancer was first reported by Groves^5^ as removal of the lymphatic tissue comprising cancer cells, including bursectomy. Microscopic studies indicate the importance of milky spots of the greater omentum for cancer cell proliferation leading to peritoneal dissemination and have concluded that the greater omentum should be removed^6–8^. Haverkamp et al*.*^9^ insisted that omentectomy has prognostic and oncologic value in the curative treatment of patients with gastric cancer. In a clinical practice, Japanese gastric cancer treatment guidelines indicate that removal of the greater omentum is usually recommended in standard gastrectomy for T3 or deeper tumours^10^. National Comprehensive Cancer Network Guidelines Version 1 mentions that D1 dissection entails resection of both the greater and lesser omenta^11^.

On the other hand, a recent randomized controlled study denies a survival advantage of bursectomy for cT3–cT4a gastric cancer patients^12^. The OMEGA trial indicated that the incidence of metastases in the greater omentum is low, and that metastases are associated with advanced disease and non-radical features^13^. One propensity-matched retrospective cohort study investigated the long-term outcomes of omentum preservation and concluded that omentum-preserving gastrectomy for advanced gastric cancer (ACG) did not affect relapse and survival rates^14^.

Thus, the efficacy of omentectomy is still controversial. To prove the long-term outcomes of omentum preservation for patients with AGC, defined as clinical T3 invasion or deeper, we conducted a retrospective analysis with propensity score matching (PSM) after multiple imputation (MI) for missing values. The 3-year overall survival rate (OS) and relapse-free survival rate (RFS) were compared as the primary endpoints to examine whether omentum preservation affects outcomes. The secondary endpoints were the complication rate, the first recurrence site.

## Results

### Baseline characteristics

The numbers of patients who met our inclusion criteria were 94 for the omentum preserved group (OPG) and 144 for the omentum resected group (ORG). The baseline characteristics of each group before PSM are summarized Table 1. The greater omentum tended to be resected in patients with more advanced clinical T and N stage. The greater omentum was resected less with laparoscopic resection. The median follow-up period was 58 months, with a range of 0–94 months.

**Table 1:** Patient characteristics and operative outcomes before and after propensity score matching with multiple imputation and complete cases.

	Before PSM			PSM with MI			PSM with CC		
	OPG	ORG	P value	OPG	ORG	P value	OPG	ORG	P value
N	94	144		73	73		70	70	
Sex, male^a^	61 (64.9)	105 (72.9)	0.24	50 (68.5)	48 (65.8)	0.86	48 (68.6)	46 (65.7)	0.86
Age^a^	67.0 (38–94)	67.0 (37–90)	0.73	67.0 (41–94)	69.0 (37–90)	0.66	66.5 (42–94)	65.0 (37–90)	0.76
NAC^a^	7 (7.4)	19 (13.2)	0.24	5 (6.8)	8 (11.0)	0.56	5 (7.1)	7 (10.0)	0.76
Conversion surgery^a^	0 (0.0)	3 (2.1)	0.42	0 (0.0)	0 (0.0)	1.00	0 (0.0)	0 (0.0)	1.00
Alb^a^	4.1 (2.4–5.2)	4.1 (2.1–5.2)	0.69	4.0 (2.4–5.2)	4.0 (2.1–4.9)	0.96	4.0 (2.4–5.2)	4.12 (2.1–4.9)	0.27
Hb^a^	12.8 (5.0–17.1)	13.0 (5.3–18.0)	0.54	12.7 (5.8–17.1)	12.5 (5.3–17.7)	0.71	12.7 (5.8–17.1)	12.5 (5.0–17.7)	0.83
BMI^a^	22.4 (16.4–32.6)	22.2 (14.1–34.3)	0.69	22.4 (16.4–32.6)	22.2 (15.5–30.0)	0.93	22.4 (16.4–32.6)	22.2 (15.8–30.3)	0.84
**ASA-PS**^**a**^									
1	11 (11.7)	10 (6.9)	0.43	6 (8.2)	7 (9.6)	0.92	6 (8.6)	6 (8.6)	1.00
2	70 (74.5)	115 (79.9)		56 (76.7)	57 (78.1)		53 (75.7)	54 (77.1)	
3	13 (13.8)	19 (13.2)		11 (5.1)	9 (12.3)		11 (15.7)	10 (14.3)	
**CCI**^**a**^									
0	68 (72.3)	107 (74.3)	0.56	54 (74.0)	58 (79.5)	0.95	51 (72.9)	54 (77.1)	1.00
1	18 (19.1)	20 (13.9)		12 (16.4)	10 (13.7)		12 (17.1)	9 (12.9)	
2	2 (2.1)	7 (4.9)		2 (2.7)	2 (2.7)		2 (2.9)	2 (2.9)	
3	4 (4.3)	4 (2.8)		3 (4.1)	2 (2.7)		3 (4.3)	3 (4.3)	
4	1 (1.1)	5 (3.5)		1 (1.4)	0 (0.0)		1 (1.4)	1 (1.4)	
5	1 (1.1)	1 (0.7)		1 (1.4)	1 (1.4)		1 (1.4)	1 (1.4)	
**cT**^**a**^									
3	47 (50.0)	51 (35.4)	< 0.05	32 (43.8)	35 (47.9)	0.74	30 (42.9)	30 (42.9)	1.00
4	47 (50.0)	93 (64.6)		41 (56.2)	38 (52.1)		40 (57.1)	40 (57.1)	
**cN**^**a**^									
0	26 (27.7)	14 (9.7)	< 0.05	13 (17.8)	13 (17.8)	0.93	13 (18.6)	11 (15.7)	0.89
1	21 (22.3)	29 (20.1)		17 (23.3)	17 (23.3)		16 (22.9)	16 (22.9)	
2	23 (24.5)	46 (31.9)		22 (30.1)	19 (26.0)		20 (28.6)	15 (40.0)	
3	24 (25.5)	55 (38.2)		21 (28.8)	24 (32.9)		21 (30.0)	28 (20.0)	
**Tumour location**^**a**^									
Lower	30 (31.9)	39 (27.1)	0.17	24 (32.9)	24 (32.9)	0.60	25 (35.7)	25 (35.7)	1.00
Middle	31 (33.0)	66 (45.8)		28 (38.4)	33 (45.2)		25 (35.7)	26 (37.1)	
Upper	33 (35.1)	39 (27.1)		21 (28.8)	16 (21.9)		20 (28.6)	19 (27.1)	
**Circumference**^**a**^									
Ant	17 (18.1)	29 (20.1)	0.32	15 (20.5)	14 (19.2)	0.944	15 (21.4)	12 (17.1)	0.98
Circ	8 (8.5)	25 (17.4)		8 (11.0)	11 (15.1)		7 (10.0)	8 (11.4)	
Gre	9 (9.6)	14 (9.7)		8 (11.0)	6 (8.2)		8 (11.4)	7 (10.0)	
Less	43 (45.7)	52 (36.1)		31 (42.5)	31 (42.5)		30 (42.9)	32 (45.7)	
Post	17 (18.1)	24 (16.7)		11 (15.1)	11 (15.1)		10 (14.3)	11 (15.7)	
**Tumour type**^**a**^									
0	11 (11.7)	11 (7.6)	0.12	6 (8.2)	7 (9.6)	0.991	5 (7.1)	6 (8.6)	0.95
1	8 (8.5)	8 (5.6)		5 (6.8)	5 (6.8)		5 (7.1)	3 (4.3)	
2	21 (22.3)	30 (20.8)		18 (24.7)	16 (21.9)		17 (24.3)	15 (21.4)	
3	50 (53.2)	74 (51.4)		40 (54.8)	40 (54.8)		40 (57.1)	43 (61.4)	
4	4 (4.3)	14 (9.7)		4 (5.5)	5 (6.8)		3 (4.3)	3 (4.3)	
5	0 (0.0)	7 (4.9)		0 (0.0)	0 (0.0)		0 (0.0)	0 (0.0)	
**Operation**^**a**^									
Total gastrectomy	42 (44.7)	60 (41.7)	0.75	29 (39.7)	26 (35.6)	0.73	26 (37.1)	25 (35.7)	1.00
**Lymphadenectomy**^**a**^									
D2 or more	70 (74.5)	127 (88.2)	< 0.05	58 (79.5)	64 (87.7)	0.26	56 (80.0)	61 (87.1)	0.36
**Other organ resection**^**a**^									
Total	10 (10.6)	44 (30.6)	< 0.05	10 (13.7)	8 (11.0)	0.80	10 (14.3)	10 (14.3)	1.00
Liver	0 (0.0)	1 (0.7)	1.00	0 (0.0)	0 (0.0)	1.00	0 (0.0)	0 (0.0)	1.00
Pancreas	7 (7.4)	9 (6.2)	0.92	7 (9.6)	3 (4.1)	0.33	7 (10.0)	1 (1.4)	0.06
Spleen	9 (9.6)	34 (23.6)	< 0.05	9 (12.3)	7 (9.6)	0.79	9 (12.9)	9 (12.9)	1.00
Transverse colon	1 (1.1)	5 (3.5)	0.46	0 (0.0)	0 (0.0)	1.00	0 (0.0)	1 (1.4)	1.00
Transverse mesocolon	0 (0.0)	3 (2.1)	0.42	1 (1.4)	1 (1.4)	1.00	1 (1.4)	0 (0.0)	1.00
Laparoscopic resection	63 (67.0)	46 (31.9)	< 0.05	47 (64.4)	30 (41.1)	< 0.05	45 (64.3)	29 (41.4)	< 0.05

Data are expressed as the median (range) for continuous variables and the number of cases (%) for categorical variables.

*PSM* propensity score matching, *MI* multiple imputation, *CC* Complete cases, *NAC* neoadjuvant chemotherapy, *Alb* serum albumin level, *Hb* serum haemoglobin level, *BMI* body mass index, *ASA-PS* American Society of Anesthesiologists Physical Status, *CCI* Charlson Comorbidity Index, *Ant* anterior, *Circ* circular, *Gre* greater curvature, *Less* lesser curvature, *Post* posterior.

^a^Indicates covariates to calculate the propensity score.

### PSM after MI

In this study, we performed two types of PSM because of missing values. Initially, MI was conducted to predict missing values, and then the cases were matched based on the propensity scores. Then, PSM was performed with complete cases to confirm the validity of PSM after MI.

**Figure 1:**
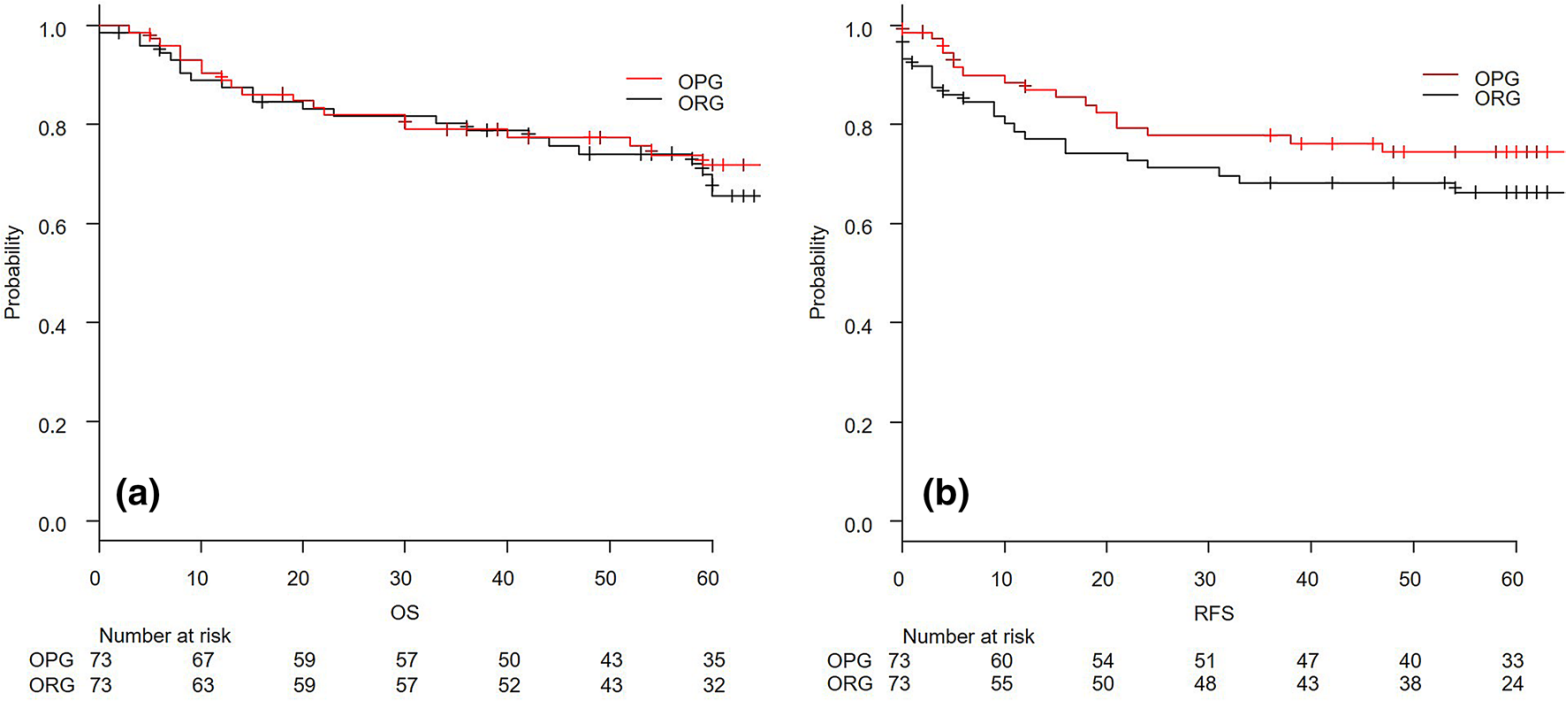
Kaplan–Meier curve of overall survival (**a**) and relapse-free survival (**b**) after propensity matching with multiple imputation. *OPG* omentum-preserved group, *ORG* omentum-resected group, *OS* overall survival, *RFS* relapse-free survival.

PSM after MI yielded 73 cases in each group without any difference in the patients’ backgrounds and operative outcomes except for the rate of laparoscopic surgery, as indicated in Table 1. Table 2 summarizes the postoperative outcomes. The pathological findings did not show any significance for pathological T stage, pathological N stage, differentiation, or tumour diameter. The follow-up period was 58 months, with a range of 0–94 months. There was no significant difference in the 3-year OS and RFS rates. The 3-year OS of the OPG and the ORG were 78.9 (67.4–86.7) % and 78.9 (67.4–86.7) %, respectively, with a P value of 0.54. The 3-year RFS rates of the OPG and the ORG were 77.8 (65.9–86.0) % and 68.2 (55.8–77.8) %, respectively, with a P value of 0.24. The survival and RFS curves are described in Fig. 1. Regarding the secondary endpoint, as summarized in Table 3, there was similarity in the ratio of any complication rate and severity. The rates of carcinomatous peritonitis and peritoneal dissemination as the first recurrence site were similar.

**Table 2:** Postoperative outcomes before and after propensity score matching with multiple imputation and complete cases.

	Before PSM			PSM with MI			PSM with CC		
	OPG	ORG	P value	OPG	ORG	P value	OPG	ORG	P value
**pT**									
0	0 (0.0)	1 (0.7)	0.54	0 (0.0)	1 (1.4)	0.90	0 (0.0)	1 (1.4)	0.78
1	11 (11.7)	10 (6.9)		7 (9.6)	6 (8.2)		6 (8.6)	5 (7.1)	
2	18 (19.1)	22 (15.3)		15 (20.5)	16 (21.9)		16 (22.9)	12 (17.1)	
3	39 (41.5)	66 (45.8)		28 (38.4)	31 (42.5)		27 (38.6)	32 (45.7)	
4	26 (27.7)	45 (31.2)		23 (31.5)	19 (26.0)		21 (30.0)	20 (28.6)	
**pN**									
0	39 (41.5)	36 (25.0)	< 0.05	31 (42.5)	22 (30.1)	0.39	29 (41.4)	22 (31.4)	0.55
1	19 (20.2)	27 (18.8)		14 (19.2)	16 (21.9)		14 (20.0)	14 (20.0)	
2	17 (18.1)	35 (24.3)		10 (13.7)	16 (21.9)		9 (12.9)	14 (20.0)	
3	19 (20.2)	46 (31.9)		18 (24.7)	19 (26.0)		18 (25.7)	20 (28.6)	
**Tumour diameter**									
mm	48 (8–140)	53 (0–230)	0.24	50 (18–140)	48 (0–230)	0.54	50.5 (18–140)	48 (0–149)	0.39
**Differentiation**									
tub	42 (44.7)	44 (30.6)	0.22	35 (47.9)	25 (34.2)	0.25	35 (50.0)	22 (31.4)	0.15
pap	3 (3.2)	5 (3.5)		1 (1.4)	1 (1.4)		1 (1.4)	2 (2.9)	
por	45 (47.9)	84 (58.3)		34 (46.6)	39 (53.4)		31 (44.3)	39 (55.7)	
sig	0 (0.0)	3 (2.1)		0 (0.0)	3 (4.1)		0 (0.0)	2 (2.9)	
muc	4 (4.3)	7 (4.9)		3 (4.1)	4 (5.5)		3 (4.3)	4 (5.7)	
None	0 (0.0)	1 (0.7)		0 (0.0)	1 (1.4)		0 (0.0)	1 (1.4)	
Adjuvant chemotherapy	56 (59.6)	103 (71.5)	0.08	45 (61.6)	49 (67.1)	0.60	44 (62.9)	52 (74.3)	0.20
**Medication**									
TS1	51 (92.2)	95 (91.1)	0.77	40 (88.9)	46 (89.8)	0.47	39 (88.6)	50 (96.2)	0.30
UFT	5 (7.8)	8 (8.9)		5 (12.1)	3 (11.2)		5 (11.4)	2 (3.8)	
**Duration**									
Month	12 (0–33)	12.0 (0–37)	0.34	12 (1–18)	12 (1–37)	0.43	12 (1–18)	12 (1–37)	0.54
**Follow up period**									
Month	60 (3–89)	55.5 (0–94)	0.48	59 (3–86)	56 (0–94)	0.71	60 (3–86)	57 (40–94)	0.81
**Death cases**									
n	22 (23.4)	52 (36.1)	0.05	19 (26.0)	23 (31.5)	0.58	17 (24.3)	26 (37.1)	0.14
**Recurrence cases**									
n	18 (19.1)	53 (36.8)	< 0.05	17 (23.3)	23 (31.5)	0.35	15 (21.4)	24 (34.3)	0.13

Data are expressed as the median (range) for continuous variables and the number of cases (%) for categorical variables.

*PSM* propensity score matching, *MI* multiple imputation, *CC* Complete cases, *tub* tubular, *pap* papillary, *por* poor differentiated, *sig* sigmoid, *muc* mucinous, *TS1* Titanium silicate 1, *UFT* uracil-tegafur.

**Table 3:** The first recurrence organs and complications before and after propensity score matching.

	PSM after MI				PSM with CC			
	OPG	ORG	P value	Odds ratio (95% CI)	OPG	ORG	P value	Odds ratio (95% CI)
**Recurrence cases**								
Total	17 (23.3)	23 (31.5)	0.35	0.65 (0.29–1.47)	15 (21.4)	24 (34.3)	0.13	0.53 (0.23–1.18)
**First recurrence organs**								
Peritoneum	9 (12.3)	6 (8.2)	0.59	1.57 (0.47–5.67)	8 (11.4)	7 (10.0)	1.00	1.16 (0.34–4.01)
Liver	3 (4.1)	0 (0.0)	0.25	NA	2 (2.9)	0 (0.0)	0.50	NA
Lymph nodes	4 (5.5)	4 (5.5)	1.00	1.00 (0.18–5.60)	4 (5.7)	5 (7.1)	0.79	0.80 (0.15–3.85)
Remnant cancer	1 (1.4)	0 (0.0)	1.00	NA	1 (1.4)	0 (0.0)	1.00	NA
Retroperitoneum	3 (4.1)	1 (1.4)	0.62	3.06(0.24–164.06)	2 (2.9)	0 (0.0)	0.50	NA
Ovary	0 (0.0)	1 (1.4)	1.00	NA	0 (0.0)	2 (2.9)	0.50	NA
Other	2 (2.7)	1 (1.4)	1.00	0.20 (0.10–121.20)	2 (2.9)	1 (1.4)	1.00	2.02 (0.10–121.35)
**Complications**								
Total	18 (24.7)	13 (17.8)	0.42	1.51 (0.63–3.68)	18 (25.7)	12 (17.1)	0.30	1.67 (0.68–4.19)
CD > 3	8 (11.0)	6 (8.2)	0.78	1.37 (0.37–5.08)	8 (11.4)	4 (5.7)	0.37	0.48 (0.10–1.89)
Abdominal abscess	9 (12.3)	5 (6.8)	0.40	1.90 (0.30–2.59)	9 (12.9)	4 (5.7)	0.24	2.42 (0.63–11.31)
Intestinal obstruction	2 (2.7)	4 (5.5)	0.68	0.49 (0.04–3.53)	2 (2.9)	4 (5.7)	0.68	0.49 (0.05–3.53)

Data are expressed as the number of cases (rate).

*CD* Clavien-Dindo classification, *PSM* propensity score matching, *MI* multiple imputation, *CC* complete cases, *CI* confidence interval.

### PSM with complete cases

**Figure 2:**
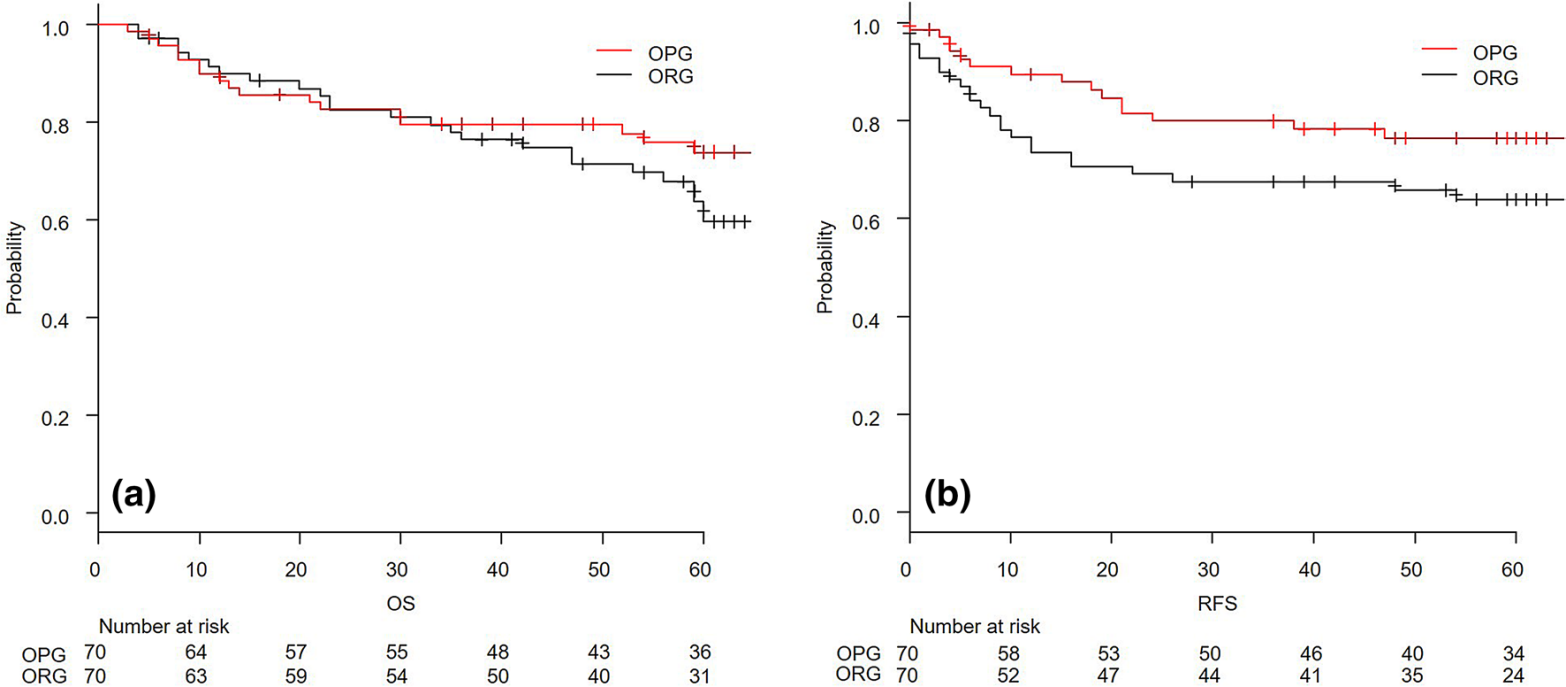
Kaplan–Meier curve of overall survival (**a**) and relapse-free survival (**b**) after propensity matching with complete cases. *OPG* omentum-preserved group, *ORG* omentum-resected group, *OS* overall survival, *RFS* relapse-free survival.

Complete cases of OPG and ORG patients were matched, and 70 patients in each group were selected for comparison. The patient background and operative and postoperative outcomes were similar to those of PSM after MI, as shown in Tables 1 and 2. The follow-up period was 59 months, with a range of 3–94 months. There was no significant difference in the 3-year OS rates of the OPG and ORG, with rates of 79.5 (67.8–87.3)% and 76.3 (64.3–84.8)%, respectively, and a P value of 0.17; the 3-year RFS rate also revealed no difference, with rates of 79.9 (67.8–87.8)% and 67.5 (55.0–77.3)%, respectively, and a P value of 0.09. The survival and RFS curves are shown in Fig. 2. In terms of the complications and the first recurrence site, there were no significant differences between the two groups (Table 3).

## Discussion

Our study indicated three important findings. First, omentum preservation for AGC patients does not affect the 3-year OS and RFS rates. Second, the ratio and severity of postoperative complications were similar in the two groups. Finally, the first recurrence site does not depend on omentum preservation. These results support our hypothesis that omentectomy could be omitted during AGC surgery in terms of short- and long-term outcomes.

Our result shows no difference in the primary and secondary endpoints with the two types of PSM, indicating that PSM after MI should be appropriate for the data. These results lead to the idea that if the tumour cells are harboured and proliferate in the greater omentum, they would not be limited to the stomach, the related lymph nodes and the greater omentum but would spread to the unresectable organs or the abdominal cavity. The result of OMEGA trial supports this theory^13^ in which resection of the greater omentum did not yield any difference in OS and RFS. In our study, there were also no differences in the first recurrence site after surgery. This finding suggests that omentectomy does not lead to a preferential pathway of metastasis. Omentum resection would be used to decide the stage of AGC but not for radicality. The existence of tumour cells in the greater omentum would be recognized as more advanced stage. However, it is impossible to prove that tumour cells do not exist in the greater omentum. Consequently, we believe that omentectomy could be omitted in terms of OS and RFS for radicality in the present treatment strategy.

The complications after gastrectomy lead to a poor long-term prognosis for OS and disease-specific mortality^15^. Although our study does not show the significance regarding the rate and site of complications, the greater omentum may play an important role in immunity and prevention of peritonitis from complications^16,17^. One study with omentum-preserving gastrectomy for early gastric cancer indicated that the group with omentum preservation had a lower rate of abdominal complications^18^. In our study, RFS of the OPG tended to be better than that in the ORG, though no significant difference was found. The reason for this might be that the preserved greater omentum reduced or minimized complications and led to better RFS. In an immunity point of view, omentum resection should be abandoned in the abdominal cavity to reduce the complication risk and future disaster in the abdominal cavity.

Finally, the contribution of postoperative adjuvant chemotherapy is also a factor to assist in omitting omental resection, which is already clear from the results of the ACTS-GC and JACCRO GC-07 studies^19,20^. There is also the possibility that adjuvant chemotherapy may provide a cure even if omental preservation has left the concerning aforementioned milky-spots. Laparoscopic surgery has also been reported to potentially be associated with shorter intervals before adjuvant chemotherapy, which can assist the effect of adjuvant chemotherapy^21^. The majority of the OPG group underwent laparoscopic surgery in our study. Most patients who were diagnosed with pathologically advanced cancer received postoperative adjuvant chemotherapy according to the guidelines^10^. There is a variation in adjuvant chemotherapy according to the guidelines of the era. However, our study did not show a significant difference between the groups, which assures that the outcomes were compared.

The limitations of this study are that, first, it was a single institutional retrospective study with a relatively small number of participants. However, the appropriate number of patients for a non-inferiority trial in a randomized controlled trial was 76 in each group^22^. This size is calculated with 80% power and a significance level of 2.5% for one side with a non-inferior margin for the difference in the survival rate, assuming non-inferiority margin, delta as 5%. The estimated 3-year OS rate of the ORG was 48.4%, and that of the OPG was 65.6% in a previous report^14^. This result indicates that our sample size was not too small to compare the outcomes. However. the 3-year OS rate was higher than the already reported and the difference between OPG and ORG was smaller than expected. Consequently, more cases are required to reveal the non- inferiority of omentum preservation. The second limitation is the diagnostic validity and eligibility criteria. We enrolled patients diagnosed with advanced gastric cancer, that is, cT3 and 4 tumours, because preoperative diagnosis of the depth of tumour invasion is difficult^23^. On the contrary, patients clinically diagnosed with cT3 and 4 include those with pathologically cT1 and 2, accounting for approximately 30% of the ratio. This may not accurately reflect the impact of omentum preservation in AGC. The last limitation is that the OPG contains more laparoscopic surgery cases than the ORG. We did not match the operation approach because there are several reports that show that laparoscopic resection does not show inferiority in long-term outcomes^24,25^. Hence, we focused on the status of the greater omentum. Our study has these limitations; therefore, it is difficult to adapt all cases. Nevertheless, our study has value for the initiation of a well-designed randomized control trial in the future.

## Conclusion

There was no significant difference in long-term outcomes between the OPG and ORG for AGC patients in our PSM study. This result indicates the possibility of the omission of omentectomy during surgery.

## Methods

### Patients and data collection

**Figure 3:**
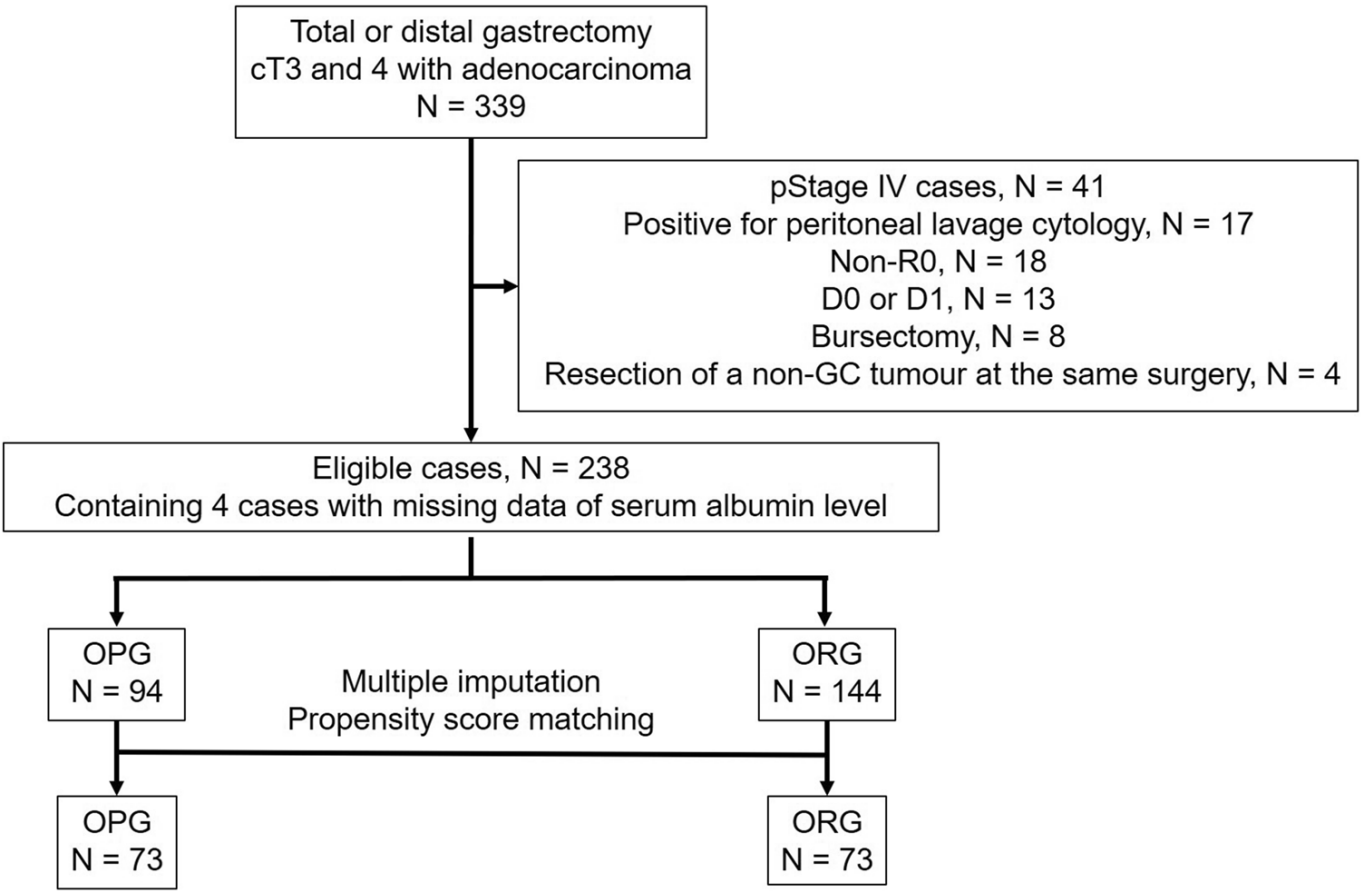
Study design. pStage pathological stage, non-GC non-gastric cancer, OPG omentum-preserved group, ORG omentum-resected group.

We identified 399 patients with cT3 and 4 adenocarcinomas who underwent either distal or total gastrectomy at Ishikawa Prefectural Central Hospital from March 2008 to August 2017 by retrospectively reviewing medical records. The cases of curability with R0 resection and D1+ , D2 or D2+ lymph node dissection was eligible. Patients who underwent bursectomy, those with M1 including positive peritoneal lavage cytology, and those who underwent resection of other organs during the same surgery due to another primary tumour were excluded. The number of eligible patients was 238, and they were divided into 2 groups, with the OPG including 94 patients, and the ORG including 144 patients. We performed multiple imputation for missing values and propensity matching to remove the biases, and 73 patients in each group were subsequently compared (Fig. 3).

### Surgical procedure

Gastrectomy was performed by board certified surgeons either laparoscopically or laparotomically. In the case of laparoscopic surgery, the qualified surgeons successfully underwent the endoscopic surgical skill qualification system in Japan. For clinically AGC, peritoneal lavage cytology was performed. When peritoneal metastasis was suspected, intraoperative biopsy was performed during surgery. Thus, accurate pathological diagnosis was determined. In OPG patients, the greater omentum was preserved, with dissection from at least 3 cm inferior to the greater curvature along with the gastroepiploic artery and vein in principle or with dissection of the sparse area of the greater omentum when the distance between the stomach and transverse colon was short. The first branch to the greater curvature of the left gastric epiploic artery and vein was resected with No.4sb lymph node dissection, preserving the branch of the greater omentum to avoid ischaemia. The root of the right gastric epiploic vessels was resected with No. 6 lymph node dissection. This procedure left most of the greater omentum in the abdominal cavity. The rest of the procedure was adequately performed in the same manner in all cases.

Historically, the greater omentum was removed when AGC was clinically suspected. Recently, however, omentectomy has been omitted except in cases where severe tumours or lymphatic invasion at the omentum was identified. Nevertheless, the final indication depended on the surgeon’s decision.

### Clinical and pathological evaluation

For the baseline characteristics, body mass index and laboratory data were obtained within 1 month before surgery from medical records. Clinical staging and tumour location were decided based on preoperative upper gastrointestinal endoscopy, enhanced computed tomography scan, fluoroscopy of the stomach, and surgical findings. The Charlson Comorbidity Index was introduced to assess the complications before surgery^26,27^. Tumour staging was based on the Union of International Cancer Control TNM classification (8th edition)^28^, and lymph node dissection was defined by the Japanese classification of gastric carcinoma (3rd English edition)^29^. The resected specimen was examined and diagnosed by at least two pathologists at our institution. To analyse the endpoints, the following definition was applied: complications within 2 months after surgery were graded by the Clavien-Dindo (CD) classification^30^. Severe complications were defined as those greater than CD grade IIIA. Ileus caused by carcinomatous peritonitis was excluded from postoperative complications.

### Follow-up

The patients visited the hospital at least 3 years after surgery to examine recurrence. Usually, enhanced computed tomography scan or abdominal ultrasound examination and blood tests including carcinoembryonic antigen and cancer antigen 19-9 were performed every 3 or 6 months. When any suspicious lesion of recurrence was found, further studies were conducted for definitive diagnosis. Adjuvant chemotherapy was introduced following the Japanese gastric cancer treatment guidelines^7^. Once any recurrence was diagnosed, the patients underwent either chemotherapy or palliative treatment depending on the patient’s condition. The outcomes were checked retrospectively with medical records.

### Statistical analysis

All statistical analyses were performed with R (The R Foundation for Statistical Computing, Vienna, Austria). The baseline characteristics were compared with the Mann–Whitney U test for continuous variables after confirmation of a non-normal distribution with the Kolmogorov–Smirnov test. Fisher’s exact test was used for categorical variates. To analyse 3-year OS and RFS, the Kaplan–Meier method and Cox proportional hazards regression were employed. To reduce the effect of bias and potential confounding, we planned PSM to compare the outcomes. However, missing data were detected in the serum albumin level before surgery in six cases (1.89% of the cases). These data were not measured accidentally. The missing values were imputed with MI, and propensity scores were estimated for matching. MI was conducted by calculating regression models including variables potentially related to the missing value and variables correlated with the outcome. The number of covariates was 68, including the results of the endpoints. Calculations were performed with the mouse package, and 30 multiply imputed datasets were created^31^. In these 30 complete datasets, the propensity score of each case was calculated and averaged^32^. PSM was conducted with the averaged propensity score with the following algorithm: 1:1 nearest-neighbour matching without replacement using a Caliper width 0.20 logit of the standard difference^33^. In this study, we chose variables from preoperative and perioperative findings that could affect outcomes. PSM was performed with the Matching package^34^. The imputation of missing data with MI is recommended to reduce bias by eliminating cases with missing values^35,36^. To make the results more solid, the outcomes of PSM with MI and PSM with complete cases were discussed under the same conditions. Two-tailed P values of < 0.05 were considered statistically significant. Continuous variables are presented as medians with maximum and minimum values. OS and RFS are described as rates with 95% confidence intervals.

### Ethics

All experimental protocols described in this study were approved by the Institutional Ethical Review Committee of Ishikawa Prefectural Central Hospital and met the Ethical Guidelines of Japan Ministry of Health, Labour and Welfare for Medical and Health Research Involving Human Subjects and conformed to the provisions of the Declaration of Helsinki. Due to the retrospective design, the Institutional Ethical Review Committee did not require informed consent, but the opt-out recruitment method was applied to provide an opportunity to decline participation to all patients. The authors declare no competing interests.

## Data Availability

The datasets generated during the current study are not publicly available to protect individual patient information, but data are available from the corresponding author on reasonable request.
